# Design-Expert^®^ Optimization of Tamoxifen-Loaded Transethosomal Gels: A Promising Transdermal System with Cytotoxicity and Stability Validation

**DOI:** 10.3390/pharmaceutics18080992

**Published:** 2026-08-11

**Authors:** Reem Abou Assi, Ahmed Bassam Farhan, Karam Abdullah Darweesh, Amira H. Hassan, Siok Yee Chan

**Affiliations:** 1 Thoughts Formulation Lab, School of Pharmaceutical Sciences, Universiti Sains Malaysia, Gelugor 11800, Malaysia; reem.ahmed@alqabas.edu.iq; 2 EDEN Research Group, Department of Pharmacy, Al-Qabas Private College, Mosul 41002, Iraq; 3 International Advisory Board, Rhein-Main Universities Alliance, 60629 Frankfurt am Main, Germany; 4 Department of Pharmaceutics, College of Pharmacy, Baghdad University, Baghdad 10071, Iraq; ahmed.b@copharm.uobaghdad.edu.iq; 5 Discipline of Pharmaceutical Technology, College of Pharmacy, Al-Kitab University, Altun Kupri, Kirkuk 36001, Iraq; karam.darweesh@uoalkitab.edu.iq; 6 Pharmaceutics and Industrial Pharmacy Department, Faculty of Pharmacy, Beni-Suef University, Beni Suef 62511, Egypt; amira.abdelatef@pharm.bsu.edu.eg

**Keywords:** tamoxifen, transdermal delivery, skin penetration, stability, IC_50_

## Abstract

**Background**: This study evaluates transdermal delivery of tamoxifen (TXN) as an alternative to the oral route of administration in treating breast cancer, which is the leading cause of cancer-related death in women globally. Oral TXN, a Class II drug, is associated with first-pass metabolism and serious side effects, including secondary cancers. Objectives: To enhance transdermal delivery, lipid-based transethosomes (TRS) were formulated using three different 2^4^ factorial designs with various non-ionic surfactants, including Tween 20^®^, Span 20^®^, and Span 80^®^. **Methods**: Optimized TRS formulations were incorporated into HPMC-based gels and characterized for morphology, drug content, pH, viscosity, spreadability, ex vivo skin penetration, and deposition. Additionally, cytotoxicity and stability were assessed. **Results**: All TXN-TRS gels were suitable for transdermal use; however, Span 20^®^-based TRS gel demonstrated the highest skin penetration (40.3 ± 1.5 µg/cm^2^), representing a 127-fold enhancement rate compared with the non-ethosomal TXN gel. In line with the enhanced penetration profile, cellular studies on MCF-7 cells showed concentration-dependent cytotoxicity, reaching 91.24 ± 1.01% inhibition at 2% *w*/*w* after 72 h, with an IC_50_ value of 0.85 ± 0.02% *w*/*w*. Stability testing showed all formulations were more stable under refrigeration than at dry room temperature storage, supporting their potential as preclinical transdermal tamoxifen delivery platforms. **Conclusions**: Span 20^®^-based TXN transethosomal gel markedly enhanced skin penetration while maintaining potent cytotoxic activity, supporting its further preclinical evaluation as a promising transdermal alternative to oral tamoxifen.

## 1. Introduction

Breast cancer (BC) caused an estimated 670,000 deaths globally in 2022. It was the most frequently diagnosed cancer among women in 157 of 185 countries and the leading cause of cancer death among women in 112 countries [1]. Estrogen, a female sex hormone, can bind to the estrogen receptors on breast cells and trigger various cellular processes [2,3]. In BC, estrogen hormone receptor-positive tumors account for roughly 70–80% of all diagnoses [4,5].

Tamoxifen (TXN), a selective estrogen receptor modulator, is widely used for the treatment and prevention of estrogen receptor (ER)-positive BC [6] and is administered orally as tablets or an oral solution. However, long-term oral TXN therapy, typically prescribed for 5–10 years, is subject to extensive hepatic metabolism and transporter-mediated disposition, contributing to interpatient variability in the systemic exposure to tamoxifen and its active metabolites [7]. Classified as Class II for biopharmaceutical properties, oral TXN is associated with significant side effects, including vein thrombosis, endometrial cancer, fatty liver disease, pulmonary embolism, and sleep disorders [8].

Transdermal drug delivery systems (TDDS) have several attractive merits and deserve further exploration. They could achieve topical administration and improve patient compliance, as well as reduce first-pass hepatic metabolism and side effects associated with systemic administration [9]. TDDS are being explored for various chronic and neurological conditions, including Parkinson’s disease [10], Alzheimer’s disease [11], and long-term hormone replacement therapy in elderly patients [12], as well as pain management [13], cardiovascular diseases [14], and oncology [15]. Lipid-based formulations, namely transethosomes (TRS), are a leading vehicle in TDDS, and their design is significantly influenced by the selection of excipients, particularly penetration (edge) enhancers [16]. TRS are an advanced form of the classical ethosomes, which combine vesicular flexibility and deeper penetration through the disruption of the stratum corneum barrier [17].

Additionally, localized TXN TDDS has been investigated via different nanocarriers, including Microneedle [18], solid lipid nanoparticles [19], bilosomes [20], lipoplex [21], ethosomes [22], nanoemulsion [23], and transfersomes [24]. A recent study reported TXN loaded in TRS composed of LS100, cholesterol, and ethanol, which achieved a 1.33-fold enhancement compared to TXN gel [25]. However, a systematic comparison of TRS stabilized by non-ionic surfactants with different hydrophilic–lipophilic balance (HLB) values has not been reported. Furthermore, the subsequent incorporation of such optimized transethosomes into a gel matrix for transdermal delivery of TXN, along with complete physicochemical characterization, cytotoxicity evaluation, and stability validation, remains unexplored.

Leveraging the advantages of non-ionic surfactants, this study hypothesizes that the incorporation of TRS in the transdermal delivery of TXN may result in the formation of highly deformable and flexible vesicles capable of penetrating the skin in a localized drug delivery manner. The selection of specific surfactants is based on their distinct HLB values and molecular structures, which are expected to differentially influence vesicle formation, bilayer packing, interfacial tension, and skin penetration mechanisms. Tween 20^®^ (HLB 16.7), a high-HLB surfactant with polyoxyethylene chains, provides steric stabilization and interacts with the aqueous microenvironment of the skin [26,27]. Span 20^®^ (HLB 8.6), an intermediate-HLB surfactant, exhibits balanced interactions with both lipid and aqueous phases, potentially optimizing lipid bilayer disruption [28,29]. Span 80^®^ (HLB 4.3), a low-HLB, unsaturated surfactant, preferentially partitions into lipid bilayers and may exhibit unique membrane-fluidizing properties due to its unsaturated fatty acid chain [30,31]. These differences in HLB and molecular structure are expected to significantly influence hydrodynamic diameter, entrapment efficiency, skin permeation, and, ultimately, therapeutic efficacy. Although TXN was loaded into various nanocarriers, a systematic comparison of TRS stabilized by these three non-ionic surfactants of different HLB values and their subsequent incorporation into a gel for transdermal delivery has not been reported.

This study aimed to explore the use of Tween 20^®^, Span 20^®^, and Span 80^®^ as permeation enhancers in transethosomal gels for the transdermal delivery of TXN. The investigation encompassed the formulation and statistical optimization of TXN-loaded TRS. The optimized formulations were subsequently incorporated into a gel base. The resulting TXN-TRS gels were evaluated for various physicochemical properties, including hydrodynamic diameter (D_h_), polydispersity index (PDI), zeta potential (ZP), pH, drug content, viscosity, and spreadability, as well as cytotoxic efficacy. To determine skin permeation efficiency, ex vivo skin permeation and deposition assessments were also performed. Short-term stability studies were conducted on the finally selected formulations.

## 2. Materials and Methods

### 2.1. Materials

Tamoxifen Citrate, purity > 98%, was obtained from Tokyo Chemical Inc., Tokyo, Japan. Phospholipon 90G^®^ (PL90G^®^), containing more than 90% soybean-derived phosphatidylcholine, was gifted from Lipoid LLC, Newark, NJ, USA. Absolute ethanol (purity > 99.5%) was sourced from Merck KGaA, Darmstadt, Germany. Tween 20^®^ (T20), Span 20^®^ (SP20), and Span 80^®^ (SP80) were purchased from R & M Chemicals, Essex, UK. Carbopol 940^®^ was gifted from Lubrizol Corporation, Wickliffe, OH, USA. Poloxamer 407^®^ was obtained from Sigma-Aldrich Co, St. Louis, MO, USA. High-molecular-weight grade Hydroxypropyl Methyl Cellulose (HPMC), METHOCEL K15M, was obtained from Colorcon located in Gallarate, Italy. Human breast cancer cell line (MCF-7) was obtained from the American Type Culture Collection (ATCC) (Manassas, VA, USA). The Roswell Park Memorial Institute (RPMI) 1640 Medium, Penicillin–Streptomycin solution (Pen-Strep) (which contains 10,000 units of penicillin and 10 mg of streptomycin), and Gibco^®^ fetal bovine serum (FBS) (New Zealand origin), along with Gibco^®^ Dulbecco’s Phosphate-Buffered Saline (1X) (DPBS), were purchased from ThermoFisher Scientific (Waltham, MA, USA). Trypsin–EDTA solution 1X (0.25% trypsin and 0.02% EDTA), dimethyl sulfoxide ≥ 99.5% (DMSO), and Thiazolyl Blue Tetrazolium Bromide 98% (MTT) were from Sigma Aldrich (St. Louis, MO, USA).

### 2.2. TRS Preparation

Transethosomal dispersions were prepared using the cold method. The organic phase was composed of PL90G^®^ (2–4% *w*/*w*) and a penetration enhancer (Tween 20^®^, Span 20^®^, or Span 80^®^, at 0.2–0.4% *w*/*w*) dissolved in ethanol (15–25% *w*/*w*). The aqueous phase consisted of distilled water (to 100% *w*/*w*). Initially, the aqueous phase was heated to 30 °C and gradually added to the organic phase with continuous stirring at 1200 rpm for 45 min at 30 °C. Following TRS formation, particle size was reduced using the extrusion technique, during which the dispersion was passed through a 220 nm pore-size Acrodisc^®^ syringe filter (PALL Life Sciences, Port Washington, NY, USA) for either 5 or 10 cycles [20]. The complete compositions of all formulations are presented in Table 1, Table 2 and Table 3.

### 2.3. Influence of Formulation Variables on TRS Properties

#### Design-Expert^®^

To assess the influence of formulation variables on TRS properties, three separate sets of 2^4^ full factorial experiments were conducted, each employing a different penetration enhancer (Supporting Information). The factorial designs systematically evaluated the effects of ethanol concentration, PL90G^®^ concentration, non-ionic surfactant concentration, and the number of extrusion cycles, together with their potential interactions, on the dependent responses listed in Supplementary Table S1. Data obtained from the experimental runs were statistically analyzed using Design-Expert^®^ software, version 13.0.13 (Stat-Ease, Inc., Minneapolis, MN, USA).

### 2.4. Morphological Properties: Hydrodynamic Diameter (D_h_), Polydispersity Index (PDI), and Zeta Potential (ZP)

D_h_ and PDI of the prepared TRS formulations were assessed using dynamic light scattering. These measurements were carried out with a Litesizer 100 instrument (Anton Paar, Graz, Austria) at a controlled temperature of 25 ± 2 °C. To ensure measurement accuracy, the samples were diluted with distilled water before analysis to achieve a suitable scattering intensity. Subsequently, the TRS formulation ZP was determined using a Zetasizer Nano ZS instrument (Malvern Instruments Ltd., Malvern, UK). All measurements were performed in triplicate (n = 3).

### 2.5. Entrapment Efficiency (EE%)

The TRS formulations were loaded with TXN by dissolving the drug in the organic phase during TRS preparation. The TXN-loaded TRS were then re-characterized to determine their D_h_, PDI, ZP, and EE%. To separate the encapsulated TXN from free TXN, the Sephadex G-25 mini-column centrifugation method was employed. *EE*% was quantified using high-performance liquid chromatography (HPLC) via the following equation:

$$EE\%=\frac{Encapsulated\;tamoxifen\;weight}{Tamoxifen\;initial\;weight}\times 100$$

### 2.6. HPLC Analysis

A specifically developed and validated HPLC method was used to determine the entrapment efficiency, assess permeation studies, and evaluate the drug content of the developed TXN-loaded formulations. Separation was performed using a Shimadzu liquid chromatography system (VP series, Kyoto, Japan) consisting of a CBM/20A system controller, LC/20AD solvent delivery pump, SPD/20A UV/VIS detector, SIL/20A autosampler, and CTO/10ASvp oven system. Shimadzu LabSolutionsVR software (version 5.30 SP1) (Kyoto, Japan) was used for data acquisition and analysis. Chromatographic separation was carried out using a stainless-steel reversed-phase ZORBAX Eclipse AgilentVR C-18 analytical column with dimensions of 150 mm × 4.6 mm ID × 5 µm. The mobile phase consisted of ACN and ammonium acetate buffer (pH 4.8) at a ratio of 70:30 (*v*/*v*%), delivered at a flow rate of 0.7 mL/min. The oven temperature was maintained at 45 °C, and detection was performed at a wavelength of 236 nm. The injection volume was 10 µL. Linearity was observed over the concentration range of 0.2–5 µg/mL, with the regression equation y = 28,318x − 539.83 (R^2^ = 0.9999) and a retention time (Rt) of 5.61 ± 0.001 min. The limits of detection and quantification were 0.027 and 0.082 µg/mL, respectively. The method met the United States Pharmacopeia (USP) system-suitability criteria, with a theoretical plate number of N > 1500, a tailing factor of T ≤ 1.5, and a resolution of Rs > 3. Samples were analyzed in triplicate. The developed method was validated, and its accuracy, precision, and reproducibility were confirmed [20].

### 2.7. TXN-TRS Gel Preparation

Different polymers, namely Carbopol 940^®^ (at 2% *w*/*w*), Poloxamer 407^®^ (at 20%, 25%, and 30% *w*/*w*), and HPMC (at 4%, 6%, and 8% *w*/*w*), were explored. The optimized TXN-loaded TRS were mixed with a pre-prepared gel base at a ratio of 3:1. For comparative purposes, a non-transethosomal TXN gel was also prepared using the same procedure, with TXN dissolved in a 25% *w*/*w* ethanolic solution. In all gel formulations, the concentration of TXN was maintained at 0.75 mg/g.

### 2.8. TXN-TRS Gels Characterization

#### 2.8.1. Determination of pH

Approximately 0.5 g of each gel was dispersed in 20 mL of distilled water and stirred for 30 min at room temperature, after which the pH was measured using a digital pH meter (FiveEasy™ F20, Mettler-Toledo International Inc., Greifensee, Switzerland). The pH meter was calibrated before use with standard buffer solutions at pH 4.01, 7.01, and 10.01 (Mettler-Toledo, Greifensee, Switzerland) at 25 °C. All measurements were performed in triplicate (n = 3).

#### 2.8.2. D_h_, PDI, and ZP

The morphological properties of TXN-TRS gels were re-evaluated using the previously described methods in Section 2.3.

#### 2.8.3. Drug Content

The TXN-TRS gel was diluted with the mobile phase to a suitable volume, sonicated for 10 min, and quantified by HPLC.

#### 2.8.4. Transmission Electron Microscopy (TEM) Imaging for Structural Confirmation

TEM was used to examine TXN-TRS formulation and gel dispersions using a LIBRA 120 instrument (Carl Zeiss Microscopy GmbH, Jena, Germany) at 120 kV, following a previously reported protocol [32].

#### 2.8.5. Rheological Behavior

The viscosity of the TXN-TRS gels was measured using a MesuLab viscometer (Model NDJ-5S, Mesu Lab Instruments Co., Ltd., Guangzhou, China) equipped with rotor No. 2. Measurements were obtained at 1.5 and 3 rpm under ambient conditions and at a relative humidity below 80%, according to a previously reported protocol [33]. Spreadability was evaluated using the parallel-plate method [34], while the spreadability factor (*Sf*) in cm^2^/g was calculated as per the equation:

$$Sf=\frac{A}{W}$$

where (*A*) is the maximum spread area in (cm^2^) and (*W*) is the total weight stressed on the gel (in grams).

### 2.9. Skin Permeation Studies

In this study, dorsal skin from male Sprague Dawley rats, aged 7–8 weeks and weighing approximately 180–200 g, was utilized. All experimental procedures were conducted in compliance with the ethical guidelines for animal handling established by Universiti Sains Malaysia (USM/IACUC/2021/(130)(1158)), 8 April 2024. The rats received a standard pellet diet (Altromin Spezialfutter GmbH & Co. KG, Lage, Germany) and tap water ad libitum. The rats were individually placed in separate polycarbonate cages (size 365 Mm × 207 Mm × 140 H) during the experiment.

#### 2.9.1. Preparation of the Skin

Rats were euthanized with an overdose of carbon dioxide (CO_2_). The dorsal skin was shaved, excised, cleaned with PBS (pH 7.4), and inspected for visible damage. Full-thickness skin samples (0.47 ± 0.02 mm) were confirmed using a Mitutoyo digimatic micrometer (Series 293, Kanagawa, Japan) for use in Franz diffusion cells.

#### 2.9.2. Skin Permeation Evaluation

Ex vivo skin permeation studies were conducted to compare TXN-loaded TRS gels with non-TRS gels, both containing equal TXN concentrations. Franz diffusion cells (PermeGear Inc., Allentown, PA, USA) with a 0.636 cm^2^ diffusion area and 5 mL PBS (pH 7.4) in the receptor chamber were used. After 30 min equilibration, 1 g of gel was applied. Samples (0.5 mL) were taken at 1, 2, 4, 6, 8, 12, and 24 h, replaced with fresh PBS, and analyzed by HPLC. Temperature was maintained at 37.0 ± 0.1 °C with stirring at 500 rpm.

#### 2.9.3. Skin Permeation Evaluation Analysis

TXN permeation was quantified by measuring its concentration (*Ct*) in the receptor medium over the set time intervals. Calculations included receptor volume (Vr = 5 mL), sample volume (*Vs* = 0.5 mL), and sample concentration (*Ci*), expressed as a cumulative amount per unit area (*Qt*/*A*, *A* = 0.636 cm^2^). Accordingly, the cumulative amount of TXN (*Qt*) was calculated from the below:

$$Qt=[Ct\;Vr+\sum_{i=1}^{t-1}Ci\;Vs]$$

The permeation flux (*Jss*, µg/cm^2^/h) was obtained from the slope of the *Qt*/*A* vs. time plot (regression analysis method) for the different formulations as follows:

$$Jss=\left[\left(\frac{dQ}{dt}\right)\right]/A$$

where (*A*) represents the effective area of the skin through which drug permeation occurs, while (*dQ*/*dt*) represents the amount of drug that passes through the skin per unit of time when in a steady state (in µg/h) [29,35]. The permeability coefficient (*Kp*) was calculated using:

$$Kp=\frac{Jss}{Cd}$$

where *Cd* refers to the concentration of TXN that has been administered to the skin in the donor compartment [30,36]. Finally, to determine the enhancement ratio (*ER*), the following equation was utilized:

$$Enhancement\;ratio\;(ER)=\frac{Jss\;of\;the\;ethosomal\;gel}{Jss\;of\;the\;non\;ethosomal\;gel}$$

where *Jss* is the steady state flux value (µg/cm^2^/h) [31,37].

#### 2.9.4. Skin Deposition

After the 24 h permeation study, residual TXN on the skin surface was removed by gentle wiping and methanol washing. The skin was then cut, sonicated for 20 min, and centrifuged at 10,000 rpm for 5 min. The supernatant was filtered through a 0.45 µm membrane [38,39], and TXN content was quantified using HPLC.

### 2.10. Cytotoxicity

#### 2.10.1. Preparation of the Cell Line

Cells in the logarithmic phase were seeded in 96-well plates at 1 × 10^3^ cells/well and incubated for 24 h. On treatment day, unloaded and loaded TRS gels were dispersed in RPMI at concentrations of 0.25, 0.5, 1, 1.5, and 2% (*w*/*w*), vortexed, filtered (0.2 µm), and added to labeled wells. Plates were incubated at 37 °C with 5% CO_2_ and 90% humidity for 72 h.

#### 2.10.2. The MTT Cytotoxicity Assay

Cell viability was evaluated using a modified MTT assay protocol [40]. After 72 h of treatment with various concentrations of unloaded and loaded TXN-TRS gels and TXN non-ethosomal gel, the medium was replaced with fresh medium. MTT solution (1 mg/mL in DPBS, 100 µL/well) was added and incubated for 4 h. Formazan crystals were dissolved in DMSO, and absorbance was read at 570 nm (reference 690 nm) using a Multiskan™ FC photometer (Thermo Fisher Scientific Oy, Vantaa, Finland). Results were reported as Mean ± SEM, with cell viability percentages calculated relative to the RPMI control using the standard equation:

$$Viability\;\%=\frac{Abs\;sample-Abs\;blank}{Abs\;control-Abs\;blank}\times 100$$

where

*Abs sample*: Absorbance of cells treated with formulations.
*Abs control*: Absorbance of cells not treated with formulations.
*Abs blank*: Absorbance of blank.

#### 2.10.3. The Half-Maximal Inhibitory Concentration

The IC50 value for each formulation was determined by plotting dose–response curves with gel concentrations (0.25, 0.5, 1, 1.5, and 2% *w*/*w*) on the *x*-axis and cell inhibition percentages on the *y*-axis.

### 2.11. Short-Term Stability Study

#### 2.11.1. Storage Conditions

TXN-TRS gels were stored in glass bottles, sealed with parafilm, and covered with aluminum foil to prevent evaporation and light exposure. The formulations were stored under two conditions: refrigerated storage at 5 ± 3 °C and dry room-temperature storage at 25 ± 2 °C. For the dry room-temperature condition, samples were kept in a desiccator containing phosphorus pentoxide to maintain 0% RH. For the refrigerated condition, formulations were stored at 5 ± 3 °C, where the humidity was approximately 50% [41,42,43]. The six-month stability study was conducted at 0, 3, and 6 months.

#### 2.11.2. Stability Evaluation Studies

At each time interval of this study, the formulations were evaluated for pH, D_h_, PDI, ZP, drug content, and rheological behavior of the aged transethosomal gels.

### 2.12. Statistical Analysis

Minitab^®^ statistical software, version 20.4 (Minitab, Inc., State College, PA, USA), was utilized to perform statistical comparisons, where *p* < 0.05 was considered statistically significant. All experiments were performed in triplicate. Data from characterization (n = 3), skin permeation (n = 3), and MTT (n = 6) were analyzed using one-way ANOVA, and Tukey’s HSD post hoc test was applied. For the stability study, post hoc Dunnett’s test was performed, considering zero month data as the control.

## 3. Results and Discussion

### 3.1. Preparation and TRS Optimization

**Figure 1:**
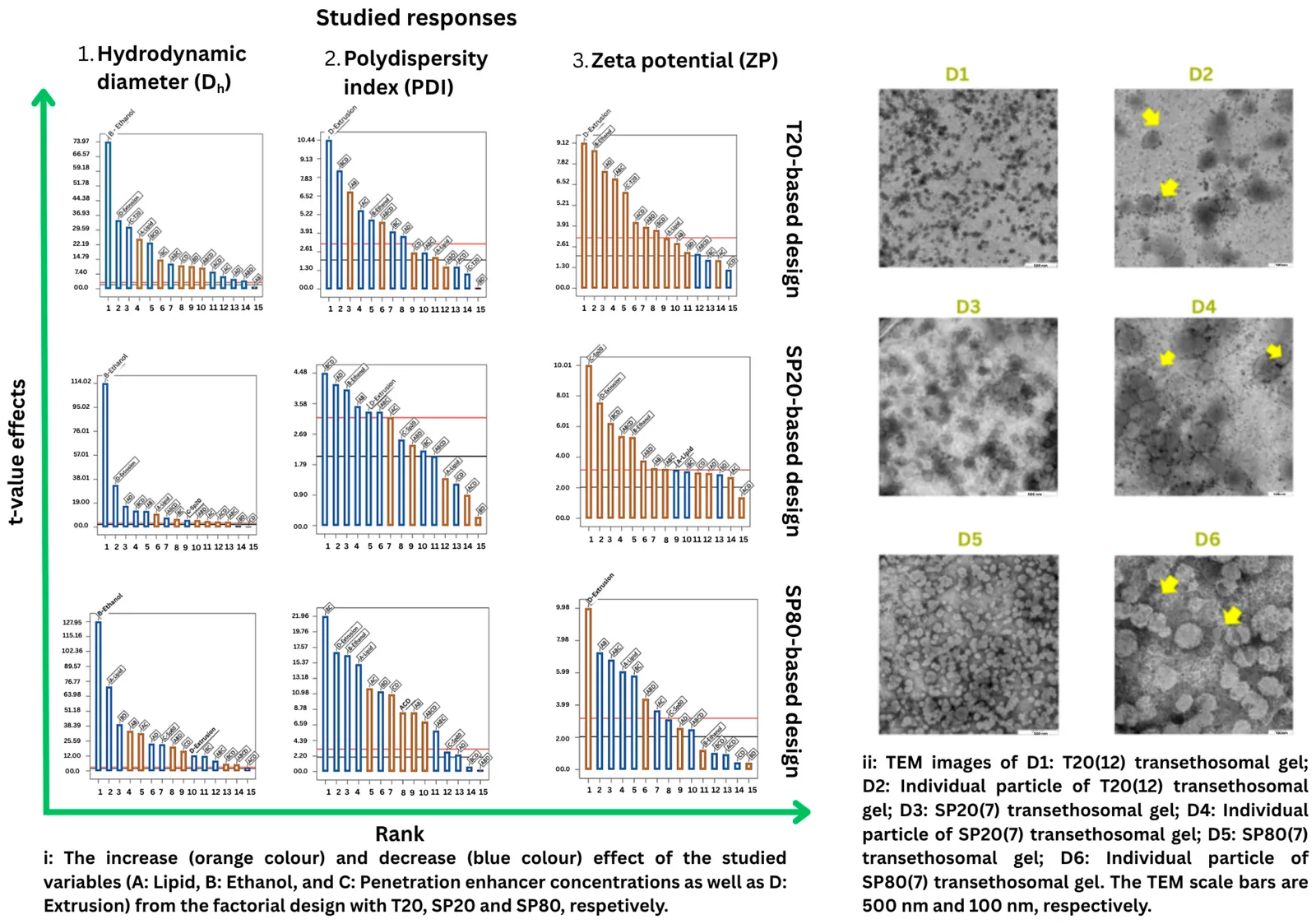
Pareto charts illustrating the process of the formulation from (**i**): factorial design 2^4^, presenting the studied factors of excipient concentrations (lipid, ethanol, and penetration enhancer) and extrusion cycles, with the responses of D_h_, PDI, and ZP to (**ii**): the TEM imaging and morphology confirmation of the different TXN-TRS gels.

The cold method was selected for TRS preparation due to its efficacy, simplicity, and scalability without harsh conditions [44]. Ethanol (10–50%) is commonly used to enhance transdermal drug delivery by disrupting skin lipid bilayers and increasing vesicle flexibility [45]. It acts as a membrane fluidizer by partitioning between phospholipid acyl chains. This disrupts hydrophobic packing and lowers the gel-to-liquid crystalline transition temperature [46]. In this study (Figure 1), ethanol concentrations of 15–25% (*w*/*w*) yielded optimal D_h_. Concentrations above 25% destabilized the vesicular membranes, whereas levels below 15% resulted in larger vesicle sizes.

PL90G^®^ (2–4% *w*/*w*) was essential for maintaining vesicle integrity; concentrations exceeding 4% led to lipid aggregation (D_h_ > 300 nm), while concentrations below 2% caused bilayer instability, particularly in the presence of high ethanol content. The incorporation of non-ionic surfactants (Tween 20^®^, Span 20^®^, and Span 80^®^ at 0.2–0.4% *w*/*w*) improved vesicular stability and produced D_h_ values below 200 nm and low PDI < 0.2. Such non-ionic surfactants (Tween/Span) contribute to this behavior due to their insertion into the phospholipid bilayer, where they reduce interfacial tension and impart steric stabilization via their hydrophilic head groups [26].

Optimal extrusion efficiency for effective skin permeation was achieved after five or ten extrusion cycles. Additional extrusion cycles showed no further improvement, aligning with the concept of the extrusion efficiency plateaus [47,48]. The formulation compositions and factorial design outcomes are summarized in Table 1, Table 2 and Table 3.

### 3.2. Analysis of the Full Factorial Designs

The fit summary (Table 4) indicates statistically significant models for all responses (*p* < 0.05), with high actual and adjusted R^2^ values (≥0.874 for all except PDI in the Sp20 design), demonstrating that most variability is explained. While most responses showed good agreement between adjusted and predicted R^2^ (difference < 0.2), the PDI response in the Sp20 design exhibited a lower predicted R^2^ (0.535) compared to its adjusted R^2^ (0.697), suggesting limited predictive capability for this response, though the model remains useful for identifying significant factors. Additionally, adequate precision values (>4) confirm a satisfactory signal-to-noise ratio and reliable model performance.

### 3.3. Effects of Formulation Variables on D_h_,PDI, and ZP

#### 3.3.1. D_h_ and PDI by Surfactant Type

The three factorial designs revealed that vesicle characteristics varied according to the surfactant type. Tween 20^®^ (HLB = 16.7) produced the smallest and most uniform vesicles (D_h_: 70.24–151 nm; PDI: 0.10–0.25). This finding may reflect its superior ability to reduce interfacial tension and promote greater bilayer curvature [27]. Span 20^®^ (HLB = 8.6) produced significantly larger vesicles (D_h_: 94.74 –172.57 nm; *p* < 0.05) with comparable size uniformity (PDI: 0.11–0.22), while Span 80^®^ (HLB = 4.3) generated the largest vesicles (D_h_: 91.48–186.74 nm; PDI: 0.12–0.24). Nevertheless, all three non-ionic surfactants successfully produced transethosomal systems.

#### 3.3.2. ZP by Surfactant Type

All transethosomal systems exhibited negative ZP values, primarily because of the phosphate groups within the PL90G^®^ structure [49]. Specifically, T20-based transethosomes displayed ZP values ranging from −10.74 to −26.06 mV; Span 20^®^-based formulations showed ZP values between −9.94 and −22.2 mV; and Span 80^®^-based formulations demonstrated ZP values ranging from −15.63 to −27.1 mV. Notably, Span 80^®^ (HLB = 4.3, unsaturated) yielded the most negative ZP, while Span 20^®^ (HLB = 8.6, saturated) produced the least negative values. Tween 20^®^ (HLB = 16.7, polyoxyethylene) showed intermediate ZP.

In this study, Span 80^®^ formulations generally exhibited the most negative ZP values, whereas Tween 20^®^ formulations showed intermediate values and Span 20^®^ formulations tended to exhibit the least negative values. These differences may be associated with variations in surfactant molecular structure, HLB, and the extent of incorporation into the phospholipid bilayer, all of which can influence the interfacial charge environment [31,50,51]. Moreover, ZP should not be considered the sole indicator of colloidal stability in systems containing non-ionic surfactants [52], as steric stabilization may also play an important role [53]. Additionally, several formulations were reported to possess stability with such ZP values, including transethosomes at −19.8 to −28.5 mV [26] and at −18.9 to −26.4 mV [54], as well as spanlastic dispersions at 18.8 to −24.5 mV [46]. These findings highlight the need for further molecular-level characterization to elucidate how surfactant orientation and phosphate-group exposure contribute to the observed ZP differences.

#### 3.3.3. Pareto Analysis of Formulation Variables

The relative influence of each formulation variable on the dependent responses was assessed using Pareto charts derived from the factorial designs (Figure 1). In TDD, D_h_ optimization is crucial as it plays a more significant role than entrapment efficiency (EE%) in achieving successful percutaneous drug transport [55]. In terms of D_h_, ethanol concentration showed the greatest size-reducing effect (*p* < 0.05), attributed to its ability to fluidize the bilayer and lower interfacial tension, promoting smaller vesicles [56]. Extrusion time was the next most influential factor (*p* < 0.05); each additional pass through the defined pore size membrane steadily decreased D_h_, in agreement with classical liposome sizing studies [47]. Surfactant concentration ranked third (*p* < 0.05), where increasing any of the non-ionic surfactant concentrations further disrupted the membrane interface, promoting smaller vesicles [57]. In contrast, increasing lipid concentration generally enlarged particles, which is consistent with other lipid-based formulations (liposome) [58], as well as transdermal vesicles utilizing PL90G^®^, oleic acid, and Labrasol (HLB ~14) as surfactants [59].

Notably, in the current study, low-HLB surfactants Span 20^®^ and Span 80^®^ exhibited a reversed lipid–D_h_ relationship where increasing PL90G^®^ concentration led to smaller vesicles. Such behavior is attributed to lipid–surfactant packing interactions, whereby the more lipophilic surfactants preferentially partition into the bilayer, promoting interfacial stabilization and increased curvature [30]. A similar trend was observed in niosome systems, where increasing cholesterol content reduced D_h_ for low-HLB surfactants (Span 60^®^ and Brij 72^®^) but not for high-HLB Tween 60^®^ [60], highlighting that low-HLB surfactants are particularly responsive to membrane-modifying components.

In terms of PDI, it was primarily influenced by extrusion cycles, which significantly narrowed the size distribution, confirming the method effectiveness and reproducibility. In contrast to the effect of PL90G^®^ concentration, increasing ethanol and surfactant levels reduced PDI, likely due to enhanced bilayer fluidity and improved mixing during vesicle formation. Similar trends have been reported for PL90G^®^-based systems using different surfactants [58] and/or liposomes prepared with Phosal 53^®^ MCT [61].

On the other hand, ZP responses followed a consistent Pareto pattern across all surfactant systems. In Tween 20^®^ stabilized transethosomes, increasing any factors (lipid, ethanol, enhancer, or extrusion) consistently reduced the magnitude of the negative ZP (typically shifting from ≈ −20 mV to −15 mV), indicating a uniform charge-shielding effect. In Span 20^®^ systems, all factors except lipid concentration made ZP less negative; increasing lipid concentrations significantly (*p* < 0.05) increased the negative charge (from −20 mV to −25 mV), likely due to tighter bilayer packing and increased surface charge density [30].

These observations suggest that high-HLB surfactants reduce measured ZP through steric shielding by their polyethylene oxide chains [50]. Formulations with low-HLB surfactants such as Span 80^®^ tend to exhibit more negative zeta potential, likely due to greater incorporation of the surfactant into the bilayer and reduced masking of surface charge groups [31]. By contrast, surfactants such as Span 20^®^, which promote tighter interfacial packing, may limit surface-charge exposure and consequently produce less negative ZP values [51]. Similar findings were reported in other lipid-based systems using Span surfactants, including Span 20^®^ and Span 80^®^ in solid lipid nanoparticles [62], Span 60^®^ in liposomes [63], and Tween 80^®^ and Span 80^®^ in transfersome design for Ibuprofen sodium salt skin delivery, where surfactant HLB and concentration were critically affecting ZP [64].

### 3.4. Tamoxifen Loading and EE%

Fifteen formulations from the three design sets met the selection criteria of D_h_ < 200 nm, PDI < 0.2, and ZP > |15 mV|. These formulations were evaluated for EE% at TXN loadings of 0.6, 1, and 1.4 mg/g, respectively. Increasing TXN loading beyond 1 mg/g led to a rise in D_h_ (still <200 nm) and a significant (*p* < 0.05) decrease in EE% to below 60%, attributed to the leaching of freely soluble TXN into the external aqueous phase [65]. Therefore, the TXN concentration of 1 mg/g was chosen for further studies. Based on the factorial optimization and entrapment efficiency, formulations T20(12), SP20(7), and SP80(7) were chosen for transethosomal gel development (Table 5).

**Figure 2:**
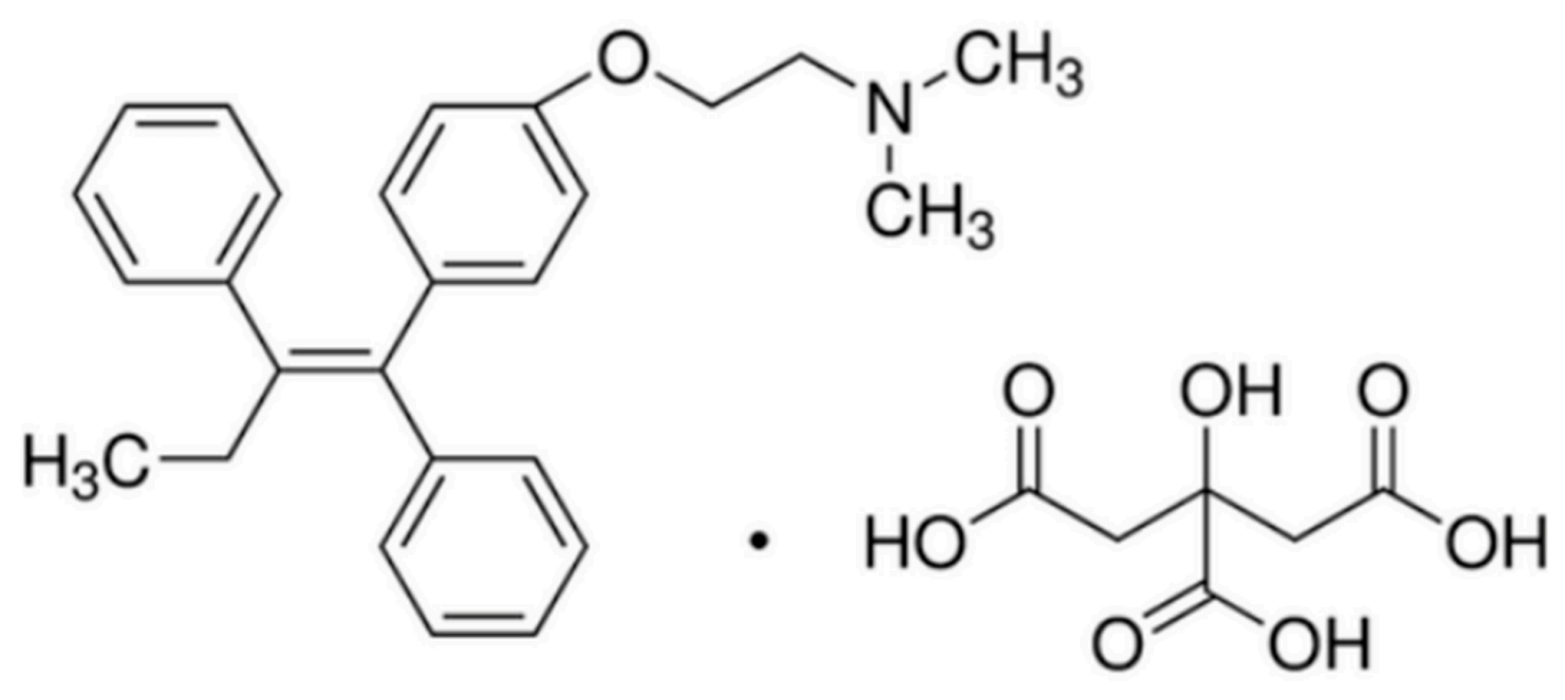
The molecular structure of tamoxifen citrate.

Importantly, upon loading TXN, ZP values shifted from negative to positive. This charge reversal is attributed to the cationic nature of tamoxifen citrate. As shown in Figure 2, TXN contains a tertiary amine group with a pKa of approximately 8.5–9.0. As per the Henderson–Hasselbalch equation, and at the formulation pH (4.94–5.11, Table 6), which is approximately 3.5–4 pH units below the pKa, the amine group is predominantly (>99.9%) protonated and positively charged [66]. The protonated drug molecules bind to the negatively charged phosphate groups of PL90G^®^ through electrostatic attraction, forming ion-pair complexes. This interaction initially neutralizes the negative surface charge and, with sufficient drug loading (1 mg/g TXN, EE% > 80%), the positive charges exceed the negative charges, resulting in net positive ZP [66,67].

### 3.5. Tamoxifen-Loaded TRS Gels Characterization

#### 3.5.1. Physicochemical Properties and Drug Content

All polymers formed transethosomal gels except Poloxamer 407^®^, which failed to produce a semi-solid structure at all studied concentrations, likely due to interference with micellar packing by ethanol or TXN, inhibiting gelation [68,69]. Carbopol 940^®^ (2% *w*/*w*) generated negatively charged transethosomes with significantly larger D_h_ > 500 nm and higher PDI (*p* < 0.05) than HPMC, likely due to its higher viscosity, which impedes uniform vesicle distribution [70]. In contrast, HPMC, a neutral polymer, produced the smallest D_h_ and PDI and preserved the positive ZP of the TXN-loaded transethosomal gel (Table 6). However, increasing HPMC concentration above 6% *w*/*w* raised viscosity, D_h_, and PDI, resulting in D_h_ levels unsuitable for transdermal delivery. Therefore, 6% *w*/*w* HPMC was selected as the optimal gel base. Cationic charge is suggested to enhance transdermal permeation due to electrostatic attraction with the negatively charged skin surface [71].

The selected transethosomal gels offered skin-friendly pH values [72,73]. Moreover, drug content values exceeded 99% of the loaded TXN concentration (0.75 mg/g), which falls within the USP-specified range of 90–110% for topical and transdermal products [74], confirming the consistency and quality of the gel formulations.

#### 3.5.2. TEM

Figure 1 demonstrates that all TXN-TRS gel formulations had a regular spherical shape, suggesting the ability of the employed non-ionic surfactants to produce such morphology, which is consistent with the D_h_ measurements obtained via the Litesizer 100 instrument (Table 6). The spherical morphology is thermodynamically favored and enables efficient encapsulation as well as uniform interaction with the skin, facilitating penetration through the stratum corneum, which is a critical requirement for effective TDD [71].

#### 3.5.3. Rheological Behavior

For effective skin penetration, generally, it is recommended that a viscosity range of 6–8 Pa·s is suitable [75,76,77]. Among transethosomal gels (Table 7), Span 80^®^ produced the highest viscosity outside the recommended range (>8 Pa·s), followed by Span 20^®^ and Tween 20^®^. All gels exhibited shear-thinning behavior, with viscosity decreasing as shear rate increased from 1.5 to 3 rpm, an essential property for topical and transdermal use [78]. Such pseudoplastic behavior is suggested to possess a controlled drug release profile in a non-Newtonian manner [75,78,79]. Spreadability significantly increased (*p* < 0.05) upon the application of the different stressing weights (Table 8). The lowest spreadability was observed in SP80(7) gel (6.77 ± 0.06 cm), while T20(12) gel showed the highest (8.03 ± 0.06 cm) (Table 7 and Table 8). All gels demonstrated a spreadability factor > 0.2, indicating desirable spreadability for transethosomal systems and efficient, uniform skin application with minimal shear [34,80].

### 3.6. Skin Permeation Evaluations

**Figure 3:**
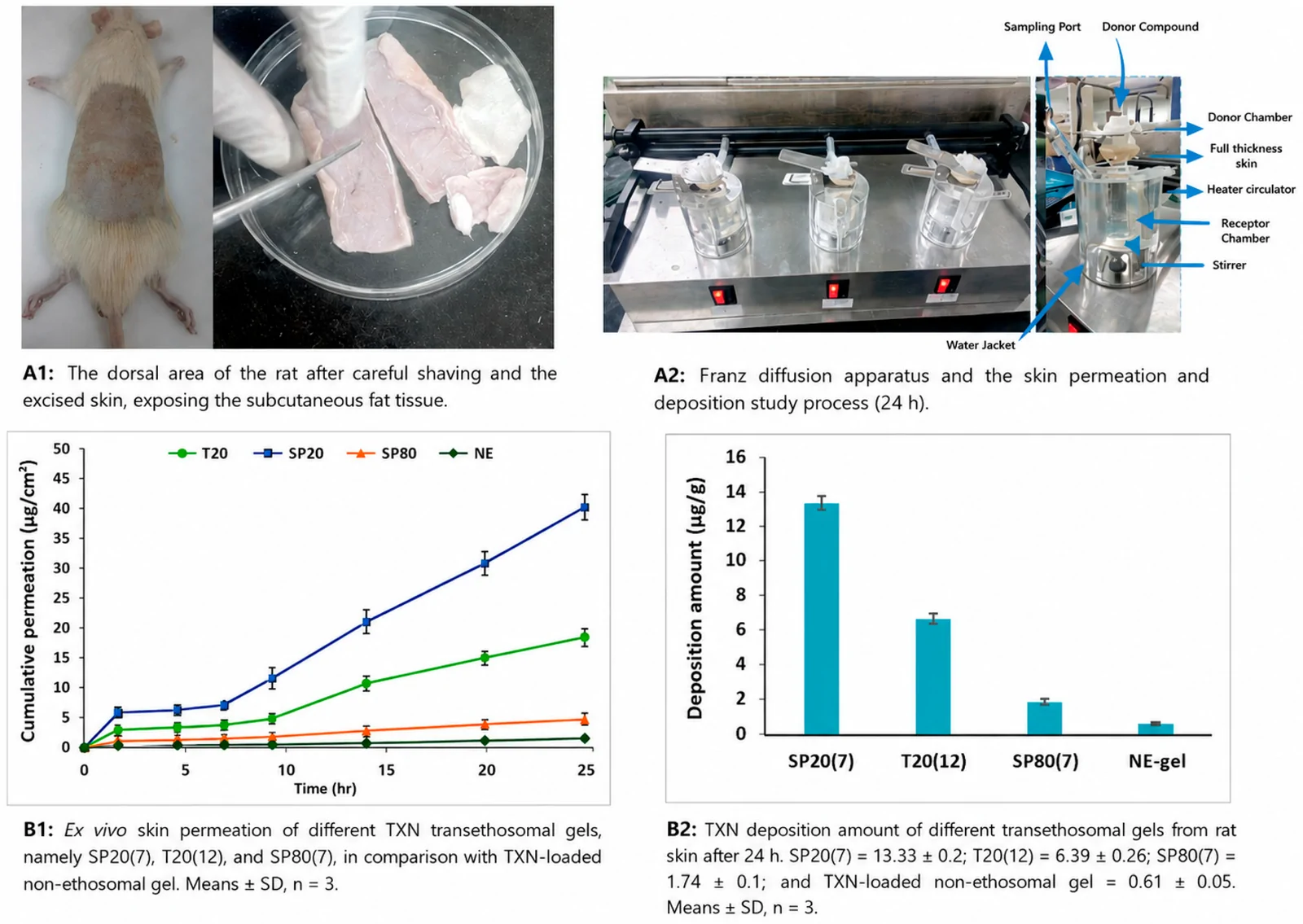
(**A1**): skin preparation for the permeation and deposition ex vivo studies, (**A2**): the process of the permeation and deposition studies via the Franz diffusion apparatus, (**B1**): the TXN-TRS gels skin permeation profile after 24 h, (**B2**): the TXN deposition from the TXN-TRS gels after 24 h.

After 24 h of permeation (Figure 3), the Span 20^®^-based transethosomal gel showed the highest transdermal delivery of TXN through full-thickness rat skin, followed by the Tween 20^®^ transethosomal gel. Although the rheological profile of the Span 80^®^-based transethosomal gel hindered TXN penetration through the skin effectively [81,82], the formulation still outperformed the non-ethosomal tamoxifen gel (NE). Skin permeation parameters are detailed in Table 9.

Several formulation-related factors may have contributed to the enhanced skin permeation of TXN. First, ethanol at concentrations of 15–25% *w*/*w* acts as a membrane fluidizer by partitioning between the polar head groups of stratum corneum lipids, lowering their melting point and increasing membrane fluidity and permeability [83,84]. Disruption of the highly organized intercellular lipid matrix may consequently create transient pathways for vesicle penetration. Second, surfactant HLB influenced permeation performance. Span 20^®^ (HLB = 8.6) produced the greatest enhancement, possibly because its intermediate lipophilicity permits effective interaction with skin lipids while maintaining adequate solubility within the formulation [28,29]. In contrast, the low flux of Span 80^®^ may be attributed to its high viscosity (13 Pa·s), which restricted drug diffusion despite its high entrapment efficiency [85,86].

The non-ionic surfactants (Span 20^®^, Tween 20^®^, and Span 80^®^) may also enhance permeation by intercalating into stratum corneum lipid bilayers, disrupting their lamellar organization, and extracting or solubilizing lipids. These effects temporarily weaken the skin barrier and facilitate deeper vesicle penetration [87,88,89,90,91].

It is important to highlight that the superior performance of TXN transethosomal gels over the previously reported TXN-bilosomal gels may stem from their differing penetration mechanisms [20]. Bilosomes rely on bile salts that interact with keratin filaments or peptide/protein components, modifying corneocyte structure and increasing permeability [92,93].

While the ex vivo rat skin model provides valuable preliminary data on formulation permeability, it is important to note that rat skin permeability is generally higher than human skin due to anatomical and physiological differences [35,36,37]. The follicular pathway is more prominent in rat skin, and the intercellular lipid organization differs, which may lead to overestimation of transdermal flux. Therefore, the absolute permeation values obtained in this study should be interpreted with caution when extrapolating to human use. However, the relative enhancement ratios and the rank order of formulation performance are likely to be maintained, as these are primarily determined by the physicochemical properties of the formulations and the mechanisms of penetration enhancement, which are consistent across species [37,38].

Although the superior permeation profiles are consistent with enhanced vesicle flexibility, they do not directly demonstrate vesicle deformability. Direct measurements, such as an extrusion-based deformability index or elasticity analysis, were not performed in the present study. Such measurements would provide additional mechanistic evidence and should be included in future investigations [16,17]. Nevertheless, the combination of ethanol (15–25% *w*/*w*) as a membrane fluidizer and non-ionic surfactants (0.2–0.4% *w*/*w*) as edge activators is well established to enhance vesicle flexibility and deformability [24,46]. These formulation characteristics distinguish transethosomes from conventional liposomes and ethosomes and may have contributed to the substantial permeation enhancement observed for the Span 20^®^-based TRS formulation.

### 3.7. Skin Deposition

All developed transethosomal gels resulted in significantly (*p* < 0.05) higher drug deposition in the full-thickness skin compared to the TXN non-transethosomal gel (Figure 2). The deposition followed the same rank order as skin permeation (Span 20^®^ > Tween 20^®^ > Span 80^®^), confirming a positive correlation between increased skin permeability and enhanced drug retention. A similar observation was also reported with other lipid-based formulations, including microneedles [94], microemulsions [95], solid lipid nanoparticles [96], and nanostructured lipid carrier gel [97].

On the other hand, although ethanol at the investigated concentrations has previously been reported to be well tolerated in transdermal formulations and the non-ionic surfactants used are generally recognized as safe for topical application, their combined effects on skin-barrier integrity and irritation require further investigation. Importantly, future studies should include comprehensive skin irritation evaluations using appropriate models, such as skin histology, transepidermal water loss (TEWL) measurements, and irritation scoring methods (e.g., Draize test) in suitable animal models or reconstructed human epidermis. These assessments will be essential to confirm the safety profile of these formulations for clinical translation.

### 3.8. Cytotoxicity

#### MTT Assay

Unloaded TRS gels exhibited high biocompatibility, with cell viability exceeding 95% at all investigated concentrations. In contrast, the TXN-TRS gels produced significant concentration-dependent inhibition of MCF-7 cells (Table 10), indicating that the observed cytotoxicity was primarily attributable to TXN. Comparable cytotoxic effects against MCF-7 cells have been reported for silver nanoparticles [98], titanium dioxide nanoparticles [99], and graphene-based nanoparticles [100].

The high viability observed following treatment with the unloaded TRS gels indicates that the carrier components were not markedly cytotoxic to MCF-7 cells under the investigated conditions. The greater inhibition produced by the TXN-loaded formulations therefore suggests that the observed cytotoxicity was primarily attributable to TXN. Although tamoxifen is a selective estrogen receptor modulator widely used against ER-positive breast cancer [6], the present findings cannot establish tumor-cell selectivity because only MCF-7 cells were evaluated. Future studies should compare the loaded and unloaded formulations in non-tumorigenic cell models, including HaCaT, HDF, and MCF-10A cells, and calculate selectivity indices.

Additionally, the cytotoxic potency of the formulations, as reflected by their IC_50_ values, was consistent with their cumulative drug permeation performance. Specifically, IC_50_ of SP20(7) was the lowest 0.85 ± 0.02 *w*/*w* (~11.3 ± 0.3 µM), followed by T20(12) (1.43 ± 0.03 *w*/*w*; ~19.0 ± 0.4 µM) and Sp80(7) (6.3 ± 0.55 *w*/*w*; ~83.9 ± 7.3 µM), respectively.

### 3.9. Stability

Stability profiles for all formulations are provided in Supplementary Tables S3–S7. Compared with refrigerated storage, dry room-temperature storage resulted in significantly greater increases in D_h_ and PDI (*p* < 0.05), possibly because of enhanced phospholipid–membrane fusion and increased bilayer fluidity at the higher temperature [32,101]. Despite these changes, all formulations maintained D_h_ < 300 nm and PDI < 0.3, remaining within the optimal range for transdermal delivery [102].

The observed stability is attributed to steric hindrance imparted by HPMC chains adsorbed on vesicle surfaces, creating repulsive barriers that prevent aggregation [100,101]. A slight but significant (*p* < 0.05) increase in ZP was noted under ambient conditions, possibly due to the presence of dissociated amino groups in tamoxifen [103,104]. This ZP trend aligns with previous studies employing HPMC in lipid-based systems [105,106,107]. The pH remained within skin-compatible values (4.9–5.1) for all formulations throughout the study. A slight but significant decrease was observed for SP20(7) and SP80(7) at 6 months under ambient conditions (*p* < 0.05). This is consistent with the previously reported ethosomal stability profile [105,106].

Rheologically, although viscosity slightly declined during storage at ambient conditions, values remained within the acceptable range (6–8 Pa·s) for a successful skin penetration [69,70]. This decline corresponds with known ageing effects on HPMC (METHOCEL K15M)-based systems, such as matrix restructuring and water loss [53]. All formulations preserved pseudoplastic (shear-thinning) behavior when the shear rate increased from 1.5 to 3 rpm, indicating retention of non-Newtonian flow properties. Additionally, the spreadability factor remained >0.2, with an inverse relationship with viscosity.

While six-month stability is adequate for early-stage development, long-term stability (12–24 months) would be required for market approval per ICH guidelines as illustrated in Section 2.11. The HPMC gel matrix and non-ionic surfactants provide steric stabilization, while the ZP contributes to electrostatic repulsion. Therefore, additional stabilizers are not considered immediately necessary. However, antioxidants such as BHT or vitamin E may further extend shelf life and should be evaluated during future formulation development.

## 4. Conclusions

This study employed three 2^4^ full factorial designs to statistically optimize transethosomes incorporating Tween 20^®^, Span 20^®^, and Span 80^®^ as surfactants and penetration enhancers. Tamoxifen-loaded transethosomes were subsequently formulated into HPMC-based gels as a non-invasive alternative to oral administration, which is associated with severe systemic side effects including thromboembolism and endometrial cancer. The Span 20^®^-based formulation emerged as the optimal system, exhibiting desirable physicochemical properties (small hydrodynamic diameter < 120 nm, high entrapment efficiency > 80%, appropriate viscosity with shear-thinning behavior, and good spreadability). Ex vivo skin permeability studies demonstrated that TXN-transethosomal gels, particularly Span 20^®^, significantly enhanced tamoxifen penetration (40.29 µg/cm^2^, 127-fold enhancement) compared to Tween 20^®^, Span 80^®^, and non-transethosomal gels. Surfactant HLB was identified as a critical determinant of permeation efficacy, with intermediate HLB (Span 20^®^, 8.6) providing optimal lipid disruption and barrier penetration. This superior skin penetration profile positively correlated with cytotoxicity against MCF-7 cells, with the Span 20^®^ formulation achieving the lowest IC_50_ (0.85% *w*/*w*). All transethosomal gels maintained acceptable stability over six months, with refrigeration (5 ± 3 °C) providing optimal preservation (diameter < 300 nm, PDI < 0.3, and drug content > 98%). These findings support transethosomal gels as a promising platform for transdermal tamoxifen delivery, offering potential to circumvent oral side effects and first-pass metabolism. Future in vivo pharmacokinetic and efficacy studies are warranted to further support its potential clinical translation.

## Figures and Tables

**Table 1:** The responses of the dependent values of D_h_, PDI, and ZP from the factorial design with independent values of A: PL90G^®^ concentration (%*w*/*w*), B: ethanol concentration (%*w*/*w*), C: Tween 20^®^ concentration (%*w*/*w*), and D: times of extrusions. Mean ± SD, n = 3.

Code	Factors				Responses		
	A	B	C	D	D_h_ (nm)	PDI	ZP (mV)
T20(1)	4	15	0.2	5	151 ± 3.6	0.2 ± 0.02	−23.3 ± 0.4
**T20(2)**	**2**	**25**	**0.4**	**5**	**84.7 ± 1.9**	**0.2 ± 0.01**	**−20.3 ± 1.2**
**T20(3)**	**2**	**25**	**0.4**	**10**	**71.1 ± 0.4**	**0.1 ± 0.01**	**−20.1 ± 0.9**
**T20(4)**	**2**	**15**	**0.2**	**10**	**101.8 ± 0.6**	**0.1 ± 0.01**	**−21.2 ± 0.2**
**T20(5)**	**4**	**25**	**0.4**	**5**	**90.7 ± 2**	**0.2 ± 0.01**	**−19.2 ± 1.4**
**T20(6)**	**2**	**15**	**0.4**	**5**	**98.3 ± 1.4**	**0.2 ± 0.01**	**−17.9 ± 0.8**
**T20(7)**	**4**	**25**	**0.2**	**10**	**92.3 ± 0.9**	**0.2 ± 0.01**	**−16.6 ± 0.7**
T20(8)	2	15	0.2	5	150.3 ± 1.4	0.2 ± 0.06	−26.1 ± 0.6
T20(9)	4	15	0.4	5	123.9 ± 1	0.2 ± 0.01	−22.9 ± 0.8
T20(10)	4	15	0.2	10	120.4 ± 0.9	0.1 ± 0.01	−17.8 ± 1.1
T20(11)	4	15	0.4	10	108.8 ± 0.9	0.1 ± 0.01	−18.9 ± 1.1
**T20(12)**	**2**	**25**	**0.2**	**10**	**76.6 ± 0.7**	**0.1 ± 0.01**	**−18.8 ± 0.5**
T20(13)	4	25	0.2	5	103.4 ± 3.1	0.3 ± 0.01	−22.7 ± 1.3
T20(14)	2	15	0.4	10	102.6 ± 2.8	0.2 ± 0.01	−20.6 ± 1.3
T20(15)	4	25	0.4	10	70.2 ± 0.6	0.1 ± 0.02	−10.7 ± 0.6
**T20(16)**	**2**	**25**	**0.2**	**5**	**77 ± 0.5**	**0.1 ± 0.01**	**−18.7 ± 0.5**

**Table 2:** The responses of the dependent values of D_h_, PDI, and ZP from the factorial design with independent values of A: PL90G^®^ concentration (%*w*/*w*), B: ethanol concentration (%*w*/*w*), C: Span 20^®^ concentration (%*w*/*w*), and D: times of extrusions. Mean ± SD, n = 3.

Code	Factors				Responses		
	A	B	C	D	D_h_ (nm)	PDI	ZP (mV)
Sp20(1)	4	15	0.2	5	172.6 ± 2.3	0.22 ± 0.01	−22.2 ± 2.9
Sp20(2)	2	25	0.4	5	111 ± 0.5	0.17 ± 0.01	−15.6 ± 0.5
**Sp20(3)**	**2**	**25**	**0.4**	**10**	**98.1 ± 0.8**	**0.14 ± 0.02**	**−15.4 ± 0.9**
Sp20(4)	2	15	0.2	10	147.5 ± 0.5	0.19 ± 0.01	−16.7 ± 0.6
Sp20(5)	4	25	0.4	5	119.1 ± 1	0.17 ± 0.02	−17.7 ± 0.9
Sp20(6)	2	15	0.4	5	145.4 ± 0.6	0.14 ± 0.01	−16 ± 0.3
**Sp20(7)**	**4**	**25**	**0.2**	**10**	**97.6 ± 0.7**	**0.17 ± 0.02**	**−19.4 ± 2**
Sp20(8)	2	15	0.2	5	150.7 ± 2.5	0.18 ± 0.02	−19.6 ± 0.4
Sp20(9)	4	15	0.4	5	158.7 ± 2.2	0.22 ± 0.02	−16.6 ± 0.7
Sp20(10)	4	15	0.2	10	141.8 ± 0.8	0.13 ± 0.02	−17.4 ± 0.4
Sp20(11)	4	15	0.4	10	146.9 ± 0.8	0.19 ± 0.03	−16.1 ± 0.4
**Sp20(12)**	**2**	**25**	**0.2**	**10**	**105 ± 1.1**	**0.18 ± 0.03**	**−16.4 ± 0.9**
Sp20(13)	4	25	0.2	5	109.1 ± 1	0.16 ± 0.01	−16.6 ± 0.7
Sp20(14)	2	15	0.4	10	142.2 ± 1.1	0.16 ± 0.02	−12.6 ± 1.5
Sp20(15)	4	25	0.4	10	94.7 ± 0.5	0.11 ± 0.01	−9.9 ± 0.4
Sp20(16)	2	25	0.2	5	110.1 ± 0.7	0.17 ± 0.02	−15.2 ± 0.5

**Table 3:** The responses of the dependent values of D_h_, PDI, and ZP from the factorial design with independent values of A: PL90G^®^ concentration (%*w*/*w*), B: ethanol concentration (%*w*/*w*), C: Span 80^®^ concentration (%*w*/*w*), and D: times of extrusions. Mean ± SD, n = 3.

Code	Factors				Responses		
	A	B	C	D	D_h_ (nm)	PDI	ZP (mV)
Sp80(1)	4	15	0.2	5	138.9 ± 1.1	0.17 ± 0.01	−22 ± 2
Sp80(2)	2	25	0.4	5	110.8 ± 1.1	0.18 ± 0.01	−18.6 ± 1.3
**Sp80(3)**	**2**	**25**	**0.4**	**10**	**97.7 ± 0.9**	**0.12 ± 0.01**	**−16.9 ± 1.2**
Sp80(4)	2	15	0.2	10	209 ± 2	0.22 ± 0.01	−18.6 ± 0.3
**Sp80(5)**	**4**	**25**	**0.4**	**5**	**104.5 ± 0.5**	**0.16 ± 0.01**	**−27.1 ± 0.7**
Sp80(6)	2	15	0.4	5	156.8 ± 2	0.23 ± 0.01	−22.3 ± 0.8
**Sp80(7)**	**4**	**25**	**0.2**	**10**	**85.4 ± 1**	**0.13 ± 0.01**	**−15.6 ± 1.3**
Sp80(8)	2	15	0.2	5	186.7 ± 2.1	0.24 ± 0.01	−22.5 ± 0.9
Sp80(9)	4	15	0.4	5	150.7 ± 1.5	0.22 ± 0.01	−20.9 ± 0.6
Sp80(10)	4	15	0.2	10	130.1 ± 1.4	0.13 ± 0.01	−20.3 ± 1
Sp80(11)	4	15	0.4	10	144.6 ± 2.2	0.23 ± 0.01	−17.8 ± 1
Sp80(12)	2	25	0.2	10	114.6 ± 1	0.19 ± 0.01	−18 ± 0.9
Sp80(13)	4	25	0.2	5	120.6 ± 0.9	0.24 ± 0.01	−22.6 ± 1.4
Sp80(14)	2	15	0.4	10	195.3 ± 1.4	0.24 ± 0.01	−19 ± 0.7
**Sp80(15)**	**4**	**25**	**0.4**	**10**	**91.5 ± 0.9**	**0.15 ± 0.01**	**−23.2 ± 0.6**
Sp80(16)	2	25	0.2	5	144.8 ± 2.4	0.23 ± 0.01	−18.4 ± 1.6

**Table 4:** Regression analysis of D_h_, PDI, and ZP responses as dependent variables for all transethosomes designs.

Design	Responses	Actual R^2^	Adjusted R^2^	Predicted R^2^	Adequate Precision
**T20**	D_h_ (nm)	0.997	0.995	0.993	82.1
	PDI	0.9177	0.878	0.814	13.81
	ZP (mV)	0.922	0.885	0.823	22.71
**Sp20**	D_h_ (nm)	0.998	0.997	0.996	98.76
	PDI	0.793	0.697	0.535	10.42
	ZP (mV)	0.914	0.874	0.808	21.67
**Sp80**	D_h_ (nm)	0.999	0.998	0.997	143.01
	PDI	0.983	0.976	0.962	30.7
	ZP (mV)	0.911	0.869	0.799	18.3

**Table 5:** TXN loaded in transethosomes at 1 mg/g characteristics. Mean ± SD, n = 3.

Code	D_h_ (nm)	PDI	ZP (mV)	EE%
T20 (3)	80.1 ± 1.1	0.15 ± 0.01	12.6 ± 3.1	77.6 ± 0.5
T20 (7)	86.6 ± 1.9	0.14 ± 0.01	6.3 ± 1.2	80 ± 0.5
**T20 (12)**	**82.4 ± 1.9**	**0.11 ± 0.01**	**8.9 ± 1.7**	**85.4 ± 0.4**
T20 (16)	105.4 ± 2.3	0.17 ± 0.01	8.8 ± 1	74.7 ± 0.4
Sp20 (3)	124.2 ± 1.6	0.15 ± 0.01	11.8 ± 1	69.4 ± 0.4
**Sp20 (7)**	**112.2 ± 2**	**0.16 ± 0.01**	**13.2 ± 1.5**	**80.2 ± 0.5**
Sp20 (12)	116.1 ± 2.1	0.14 ± 0.01	14 ± 1	78.6 ± 0.4
Sp80 (3)	109.8 ± 1.6	0.11 ± 0.01	20 ± 1.1	89.2 ± 0.4
**Sp80 (7)**	**113.3 ± 2.1**	**0.06 ± 0.02**	**19.5 ± 2.7**	**97.3 ± 0.2**
Sp80 (15)	110 ± 1.6	0.1 ± 0.01	17.3 ± 1.6	83.4 ± 0.3

**Table 6:** Characterization of various TXN-loaded transethosomal gels. Mean ± SD, n = 3.

Formulation Code	D_h_ (nm)	PDI	ZP (mV)	pH	Drug Content (%)
T20(12)	112.6 ± 1.6	0.24 ± 0.01	6.6 ± 0.7	4.9 ± 0.01	99.9 ± 0.1
SP20(7)	114 ± 1.8	0.24 ± 0.01	11.6 ± 1.1	5.1 ± 0.01	99.4 ± 0.3
SP80(7)	118.8 ± 1.7	0.18 ± 0.03	10.9 ± 0.6	5.1 ± 0.01	99.9 ± 0.6

**Table 7:** Viscosity and torque measurements at different round per minute (rpm) for the different transethosomes gel designs. Mean ± SD (n = 3).

Formulation Code	1.5 rpm		3 rpm	
	Viscosity (Pa.s)	Torque (%)	Viscosity (Pa.s)	Torque (%)
T20(12)	7.4 ± 0.01	36.7 ± 0.6	6.7 ± 0.02	66.8 ± 0.2
SP20(7)	8.4 ± 0.01	41.6 ± 0.1	8 ± 0.02	81.2 ± 0.1
SP80(7)	13.6 ± 0.1	58.6 ± 0.1	11.6 ± 0.1	89.9 ± 0.4

**Table 8:** Spreadability measurements and spread factors for the different transethosomes gel designs. Mean ± SD (n = 3).

Formulation Code	Stressing Standard Weights—Spreadability (cm)							Spread Factor (cm^2^/g)
	0 g	50 g	100 g	150 g	200 g	250 g	500 g	
T20(12)	5.2 ± 0.1	6 ± 0.04	6.6 ± 0.1	6.9 ± 0.1	7.3 ± 0.1	7.6 ± 0.1	8 ± 0.1	0.4 ± 0.01
SP20(7)	4.8 ± 0.1	5.8 ± 0.1	6.1 ± 0.1	6.6 ± 0.1	6.9 ± 0.04	7.2 ± 0.1	7.5 ± 0.04	0.4 ± 0.01
SP80(7)	4.3 ± 0.1	5.2 ± 0.1	5.6 ± 0.1	5.8 ± 0.1	6.4 ± 0.1	6.5 ± 0.04	6.8 ± 0.1	0.3 ± 0.01

**Table 9:** The cumulative tamoxifen permeated after 24 h (µg/cm^2^), the flux at steady state (*Jss*), permeability coefficient (*Kp*), and enhancement rate of different gels of transethosomal systems through rat skin. Mean ± standard deviation, (n = 3).

Formula Code	Cumulative Tamoxifen Permeated (µg/cm^2^)	*Jss* (µg/cm^2^/h)	*Kp* (cm/h)	Enhancement Ratio (*ER*)
T20(12)	18 ± 1.1	1.3 ± 0.1	0.002 ± 9.77455 × 10^−5^	56.5
SP20(7)	40.3 ± 1.5	3 ± 0.14	0.004 ± 0.0002	127.9
SP80(7)	3.2 ± 0.8	0.2 ± 0.1	0.0003 ± 8.86317 × 10^−5^	8.9
NE gel	0.9 ± 0.04	0.02 ± 0.003	3.08176 × 10^−5^ ± 4.33207 × 10^−6^	

**Table 10:** The cell inhibition percentage of TXN-transethosomal gels at the different concentrations of 0.25% *w*/*w*, 0.5% *w*/*w*, 1% *w*/*w*, 1.5% *w*/*w*, and 2% *w*/*w* and after 72 h of incubation, respectively. Mean ± SEM, n = 6.

Formulation Code	The MCF-7 Cell Inhibition Percentage of Loaded Transethosomal Gels				
	0.25% *w*/*w*	0.5% *w*/*w*	1% *w*/*w*	1.5% *w*/*w*	2% *w*/*w*
T20 (12)	7.2± 0.4	18.8± 0.8	40.9± 0.7	56.7± 1.2	63.9± 0.7
Sp20(7)	30.4± 1	41.2± 0.8	62± 1.3	82.2± 0.8	91.2± 1
Sp80(7)	1.7± 0.6	3.4± 0.9	7.1± 0.7	11.4± 1.5	15.9± 1.1
Non-ethosomal gel	22.1± 1.2	38.3± 1.3	51± 1.4	67.9± 1.2	82.2± 1.3

## Data Availability

The original contributions presented in this study are included in the article/Supplementary Material. Further inquiries can be directed to the corresponding author.

## Supplementary Materials

The following supporting information can be downloaded at: https://www.mdpi.com/article/10.3390/pharmaceutics18080992/s1, Table S1: The full three sets of factorial designs for the different transethosomal systems, using different penetration enhancers including Tween 20^®^, Span 20^®^, and Span 80^®^, respectively. Table S2: Composition of the gel base formulation. Table S3: The hydrodynamic diameter (D_h_), Polydispersity Index (PDI) and zeta potential (ZP) values of the various transethosomal gel formulations at zero timing and after three and six months of stability studies under different storage conditions of 5 ± 3 °C and 25 °C ± 2 °C respectively. Mean ± SD, n = 3. Table S4: The pH values of the various transethosomal gel formulations at zero timing and after three and six months of stability studies under different storage conditions of 5 ± 3 °C and 25 °C ± 2 °C, respectively. Mean ± SD, n = 3. Table S5: The drug content values of the various transethosomal gel formulations at zero timing and after three and six months of stability studies under different storage conditions of 5 ± 3 °C and 25 °C ± 2 °C, respectively. Mean ± SD, n = 3. Table S6: The viscosity and torque values of the various transethosomal gel formulations at zero timing and after three and six months of stability studies under different storage conditions of 5 ± 3 °C and 25 °C ± 2 °C, respectively. Mean ± SD, n = 3. Table S7: Spreadability values and spread factors of the various gels of transethosomal systems at zero timing and after three and six months of stability studies under different storage conditions of 5 ± 3 °C and 25 °C ± 2 °C, respectively. Mean ± SD, n = 3.

## Abbreviations

**Abbreviation**	**Full name**
BC	Breast Cancer
DLS	Dynamic Light Scattering
DMSO	Dimethyl Sulfoxide
DPBS	Dulbecco’s Phosphate-Buffered Saline
EE%	Entrapment Efficiency
ER	Enhancement Ratio
FBS	Fetal Bovine Serum
HLB	Hydrophilic-Lipophilic Balance
HPMC	Hydroxypropyl Methyl Cellulose
HPLC	High-Performance Liquid Chromatography
IC_50_	Half-Maximal Inhibitory Concentration
Jss	Steady-State Flux
Kp	Permeability Coefficient
MCF-7	Human Breast Cancer Cell Line
MTT	Thiazolyl Blue Tetrazolium Bromide
NE	Non-Ethosomal
PL90G^®^	Phospholipon 90G^®^
RPMI	Roswell Park Memorial Institute Medium
SEM	Standard Error of the Mean
SP20	Span 20^®^
SP80	Span 80^®^
T20	Tween 20^®^
TDDS	Transdermal Drug Delivery Systems
TEM	Transmission Electron Microscopy
TEWL	Transepidermal Water Loss
TRS	Transethosomes
TXN	Tamoxifen
D_h_	Hydrodynamic Diameter
PDI	Polydispersity Index
ZP	Zeta Potential
