# Supplementary material for: Design-Expert® Optimization of Tamoxifen-Loaded Transethosomal Gels: A Promising Transdermal System with Cytotoxicity and Stability Validation
Source: Pharmaceutics. 2026 Aug 11;18(8):992. doi: 10.3390/pharmaceutics18080992 (PMC13517008; doi:10.3390/pharmaceutics18080992)
Supplement: Supplementary file 1 [file pharmaceutics-18-00992-s001.zip › pharmaceutics-4415755-supplementary.pdf]

Design-Expert® Optimization of Tamoxifen-Loaded Transethosomal Gels: A Promising Transdermal System with Cytotoxicity and Stability Validation

Table S1: The full three sets of factorial designs for the different transethosomal systems, using different penetration enhancers including Tween 20®, Span 20®, and Span 80®, respectively.

Independent variables			Values	
	Unit	Code	Low	High
PL90G® concentration	(%w/w)	A	2	4
Ethanol concentration	(%w/w)	B	15	25
Penetration enhancer	(%w/w)	C	0.2	0.4
Number of extrusions	Times	D	5	10
**Dependent variables**				
hydrodynamic diameter (D_h) (nm)				
Polydispersity Index (PDI)				
Zeta potential (mV)				

Table S2: Composition of the gel base formulation.

Ingredients	Role	Concentration
HPMC	Gel base	6 %w/w
Methyl paraben	Preservative	0.02 %w/w
Propyl paraben	Preservative	0.01 %w/w
Propylene glycol	Solubilizer	1 %w/w
Filtered distilled water	-	Up to 100 %w/w

Table S3: The hydrodynamic diameter (D_h), Polydispersity Index (PDI) and zeta potential (ZP) values of the various transethosomal gel formulations at zero timing and after three and six months of stability studies under different storage conditions of 5 ± 3 °C and 25°C ± 2°C respectively. Mean±SD, n = 3.

**Formulation Code**	**Fresh Samples (Control) (A)**					
	**D_h (nm)**		**PDI**		**ZP (mV)**	
T20(12)	112.6± 1.6		0.24± 0.01		6.6± 0.7	
SP20(7)	114± 1.8		0.24± 0.01		11.6± 1.1	
SP80(7)	118.8± 1.7		0.18± 0.03		10.9± 0.6	
	**Refrigeration (5 ± 3 °C)**			**Dry Room Temperature (25°C ± 2°C)**		
	**3 Months (B)**					
	**D_h (nm)**	**PDI**	**ZP (mV)**	**D_h (nm)**	**PDI**	**ZP (mV)**
T20(12)	118.1± 0.7*	0.2± 0.01	7.1± 0.2	123± 0.5*	0.23± 0.01	8± 0.4*
SP20(7)	122.3± 0.9*	0.2± 0.01	11.8± 0.5	125.2± 0.7*	0.24± 0.01	13.1± 1
SP80(7)	124.8± 0.9*	0.2± 0.01*	11.6± 1.1	132.5± 0.7*	0.23± 0.01*	11.9± 0.5
	**6 Months (C)**					
	**D_h (nm)**	**PDI**	**ZP (mV)**	**D_h (nm)**	**PDI**	**ZP (mV)**
T20(12)	135.2± 0.8**	0.25± 0.01	8.2± 0.35**	145.7± 0.49**	0.27± 0.01**	8.4± 0.5**
SP20(7)	142.1± 0.7**	0.25± 0.01	12.9± 0.28	150.6± 0.58**	0.28± 0.01**	13.9± 0.1**
SP80(7)	156.4± 0.7**	0.26± 0.01**	12.5± 0.49	161.7± 0.52**	0.29± 0.01**	13.9± 0.7**

** Dunnett's test $p < 0.05$ for A & B, and ** is Dunnett's test $p < 0.05$ for A & C.*

Table S4: The pH values of the various transethosomal gel formulations at zero timing and after three and six months of stability studies under different storage conditions of 5 ± 3 °C and 25°C ± 2°C, respectively. Mean±SD, n = 3.

**Formulation Code**	**Zero time (A)**	**3 Months (B)**		**6 Months (C)**	
		**5 ± 3 °C**	**25°C ± 2°C**	**5 ± 3 °C**	**25°C ± 2°C**
	**pH**	**pH**		**pH**	
T20(12)	4.9± 0.01	4.9± 0.01	4.9± 0.03	4.9± 0.06	4.9± 0.01**
SP20(7)	5± 0.01	5.1± 0.02	5± 0.01*	5± 0.05**	4.9± 0.02**
SP80(7)	5.1± 0.01	5.1± 0.02	5.1± 0.01	5.1± 0.01**	4.9± 0.02**

**Dunnett's test $p < 0.05$ for A & B, and ** is Dunnet's test $p < 0.05$ for A & C.*

Table S5: The drug content values of the various transethosomal gel formulations at zero timing and after three and six months of stability studies under different storage conditions of 5 ± 3 °C and 25°C ± 2°C, respectively. Mean±SD, n = 3.

**Formulation Code**	**Zero time (A)**	**3 Months (B)**		**6 Months (C)**	
		**5 ± 3 °C**	**25°C ± 2°C**	**5 ± 3 °C**	**25°C ± 2°C**
	**Drug content**	**Drug content**		**Drug content**	
T20(12)	99.9± 0.1	99.8± 0.1	99.3± 0.5	99.5± 0.6	98.5± 0.4**
SP20(7)	99.4± 0.3	99.4± 0.3	99.4±0.6	99.4± 0.6	98.3± 0.9**
SP80(7)	99.9± 0.6	99.8± 0.9	99.4± 0.9	99.6± 0.8	98.4± 0.7**

** Dunnett's test $p < 0.05$ for A & B, and ** is Dunnett's test $p < 0.05$ for A & C.*

Table S6: The viscosity and torque values of the various transethosomal gel formulations at zero timing and after three and six months of stability studies under different storage conditions of 5 ± 3 °C and 25°C ± 2°C, respectively. Mean±SD, n = 3.

**Formulation Code**	**Fresh samples (Control) (A)**			
	**Viscosity (Pa.s) at 1.5 rpm**	**Torque (%) at 1.5 rpm**	**Viscosity (Pa.s) at 3 rpm**	**Torque (%) at 3 rpm**
T20(12)	7.4± 0.01	36.7± 0.6	6.7± 0.02	66.8± 0.2
SP20(7)	8.4± 0.01	41.6± 0.1	8.± 0.02	81.2± 0.1
SP80(7)	13.6± 0.1	58.6± 0.1	11.6± 0.1	89.9± 0.4

	**3 Months (B)**							
	**Refrigeration (5 ± 3 °C)**				**Dry Room Temperature (25°C ± 2°C)**			
	**1.5 rpm**		**3 rpm**		**1.5 rpm**		**3 rpm**	
	Viscosity (Pa.s)	Torque (%)	Viscosity (Pa.s)	Torque (%)	Viscosity (Pa.s)	Torque (%)	Viscosity (Pa.s)	Torque (%)
T20(12)	7.5± 0.03	35.7± 0.4	6.8± 0.1	66.1± 0.7	7.2± 0.1*	37.9± 0.3*	6.6± 0.1	66.1± 0.7
SP20(7)	8.4± 0.03	42.2± 0.6	7.9± 0.03	81.2± 0.7	8.2± 0.1*	42± 0.2	7.9± 0.1*	82.2± 0.9
SP80(7)	13.5± 0.2	61.6± 0.4*	11.5± 0.1	89.3± 0.7	12.8± 0.1*	60.7± 1.1*	11.1± 0.1*	89.2± 0.6

	**6 Months (C)**							
	**Refrigeration (5 ± 3 °C)**				**Dry Room Temperature (25°C ± 2°C)**			
	**1.5 rpm**		**3 rpm**		**1.5 rpm**		**3 rpm**	
	Viscosity (Pa.s)	Torque (%)	Viscosity (Pa.s)	Torque (%)	Viscosity (Pa.s)	Torque (%)	Viscosity (Pa.s)	Torque (%)
T20(12)	7.4± 0.03	36.4± 0.6	6.3± 0.1**	67± 0.4	7.1± 0.03**	38.7± 0.5**	6.5± 0.1**	66.5± 0.3
SP20(7)	8.4± 0.04	41.9± 0.4	8.1± 0.1	81± 0.8	8.1± 0.02**	40.8± 0.6	7.7± 0.1**	83± 0.9**
SP80(7)	13.2± 0.2	60± 0.5**	11.2± 0.3	89.8± 0.1	12.2± 0.1**	56.6± 0.7**	10.9± 0.1**	89.8± 0.1

** Dunnett's test $p < 0.05$ for A & B, and ** is Dunnett's test $p < 0.05$ for A & C.*

Table S7: Spreadability values and spread factors of the various gels of transethosomal systems at zero timing and after three and six months of stability studies under different storage conditions of 5 ± 3 °C and 25°C ± 2°C, respectively. Mean±SD, n = 3.

Formulation Code	Zero time (A)		3 Months (B)				6 Months (C)			
			5 ± 3 °C		25°C ± 2°C		5 ± 3 °C		25°C ± 2°C	
	Spreadability (cm)	Spread factor (cm^2/g)	Spreadability (cm)	Spread factor (cm^2/g)	Spreadability (cm)	Spread factor (cm^2/g)	Spreadability (cm)	Spread factor (cm^2/g)	Spreadability (cm)	Spread factor (cm^2/g)
T20(12)	8± 0.1	0.4± 0.01	8.1± 0.04*	0.4± 0.004*	8.2± 0.04*	0.4± 0.004*	8.2± 0.01**	0.4± 0.001**	8.8± 0.02**	0.5± 0.002**
SP20(7)	7.5± 0.04	0.4± 0.01	7.7± 0.03*	0.4± 0.003*	7.8± 0.01*	0.4± 0.001*	7.7± 0.01**	0.4± 0.001 **	7.9± 0.02**	0.4± 0.002**
SP80(7)	6.8± 0.1	0.3± 0.01	6.8± 0.03	0.3± 0.003	6.9± 0.01*	0.3± 0.001*	7.2± 0.06**	0.3± 0.01**	7.6± 0.01**	0.4± 0.001**

**Dunnett's test $p < 0.05$ for A & B, and ** is Dunnett's test $p < 0.05$ for A & C*
